# Immunological Separation of Bioactive Natural Compounds from Crude Drug Extract and Its Application for Cell-Based Studies

**DOI:** 10.3390/antib10040048

**Published:** 2021-12-06

**Authors:** Takuhiro Uto, Tomoe Ohta, Shunsuke Fujii, Yukihiro Shoyama

**Affiliations:** 1 Graduate School of Pharmaceutical Sciences, Nagasaki International University, 2825-7 Huis Ten Bosch-Cho, Sasebo 859-3298, Japan; ohta@niu.ac.jp (T.O.); shoyama@niu.ac.jp (Y.S.); 2 Faculty of Health Management, Nagasaki International University, 2825-7 Huis Ten Bosch-Cho, Sasebo 859-3298, Japan; fujii@niu.ac.jp

**Keywords:** monoclonal antibody, immunoaffinity column, natural compound, one-step separation method, crude extract, knockout extract, ginseng, ginsenoside, licorice, glycyrrhizin

## Abstract

In this study, we present a review on a useful approach, namely, immunoaffinity column coupled with monoclonal antibodies (MAbs), to separate natural compounds and its application for cell-based studies. The immunoaffinity column aids in separating the specific target compound from the crude extract. The column capacity was stable even after more than 10 purification cycles of use under the same conditions. After applying the crude extract to the column, the column was washed with washing buffer and eluted with elution buffer. The elution fraction contained the target compound bound to MAb, whereas the washing fraction was the crude extract, which contained all compounds except a group of target compounds; therefore, the washing fraction was referred to as a knockout (KO) crude extract. Cell-based studies using the KO extract revealed the actual effects of the natural compounds in the crude extract. One-step separation of natural compounds using the immunoaffinity column coupled with MAbs may help in determining the potential functions of natural compounds in crude extracts.

## 1. Introduction

Since ancient times, herbal medicines have been widely used in many Asian countries. Despite the great advances observed in modern medicine in recent decades, herbal medicines continue to make an important contribution to healthcare worldwide. The World Health Organization (WHO) has stated that herbal medicines are still the primary healthcare system for about 80% of the world’s population, especially in developing countries [1]. Based on the importance of herbal medicines, analytical methods for bioactive natural compounds have been developed. Technical advances in instrumental analysis have allowed the detection of a wide variety of natural compounds using highly sensitive and selective methods. In addition, molecular approaches have revealed the mechanism of action of natural compounds through both in vivo and in vitro studies.

Monoclonal antibodies (MAbs) targeting high-molecular-weight molecules, such as proteins and peptides, have been widely used since 1975 [2]. A few decades ago, there were few MAbs targeting small-molecular-weight compounds, including natural compounds and synthetic drugs. However, the number of studies on MAbs against natural compounds has increased since the 1990s. Recently, a variety of MAbs against natural compounds have been developed, and some of them are commercially available. Our previous studies have produced more than 40 kinds of MAbs targeting natural compounds and have reported multiple applications based on these MAbs. Table 1 lists representative examples of MAbs and their applications in our studies and those of others.

Enzyme-linked immunosorbent assay (ELISA) with MAbs targeting natural compounds is useful for the quality control of natural products because of their high sensitivity. Moreover, ELISA listed in Table 1 is rapid and does not require pretreatment and organic solvent when compared to other analytical methods, such as high-performance liquid chromatography (HPLC) and HPLC–mass spectrometry. We also developed “Eastern blotting”, which is an on-membrane quantitative analytical system, using MAbs against natural compounds [13,14,18,23,24,25,30,39,46,50]. Eastern blotting is a unique immunostaining method for detecting target natural compounds on a membrane, which has fixed natural compounds transferred from TLC. Furthermore, we applied MAb for the molecular breeding of medicinal plants. In the first step, a recombinant single-chain fragment variable (scFv) antibody was constructed from hybridoma cells expressing MAb against natural compounds. The scFv antibody gene was introduced into the host plant. The scFv antibody was found to express in transgenic plants and activate the biosynthesis of secondary metabolites. This plant breeding methodology can be used as a potential tool to enhance bioactive compounds [42,48].

Affinity chromatography is one of the most diverse and powerful chromatographic methods for selective purification of a molecule or group of molecules from complex mixtures. For example, cell membrane chromatography is an efficient method for the detection of bioactive components acting on a specific receptor from a complex biological system [63]. One of the applications of MAbs against natural compounds is immunoaffinity purification and separation. Immunoaffinity purification using MAb is a useful methodology for purifying larger molecules, such as proteins and peptides. However, there are only a few studies on immunoaffinity purification targeting small-molecular-weight compounds, such as natural compounds. An immunoaffinity column with MAb targeting natural compounds has made it possible to carry out single-step purification and separation of target compounds from crude drug extract. We have developed immunoaffinity columns against forskolin [10], glycyrrhizin [15], ginsenoside Rb1 [26], and solamargine [47]. Additionally, other groups have demonstrated immunoaffinity columns against puerarin [60], daizin [61], and naringin [62]. In this review, we introduce the preparation of an immunoaffinity column with MAbs targeting natural compounds and carry out one-step separation of natural compounds from crude extract using the column. Furthermore, we describe the application of this approach for cell-based studies that use fractions, which are prepared by eliminating one target compound from the crude extract using the immunoaffinity column.

## 2. Preparation of the Immunoaffinity Column with MAbs against Bioactive Natural Compounds and One-Step Separation of Natural Compounds from Crude Extract Using the Column

**Figure 1:**
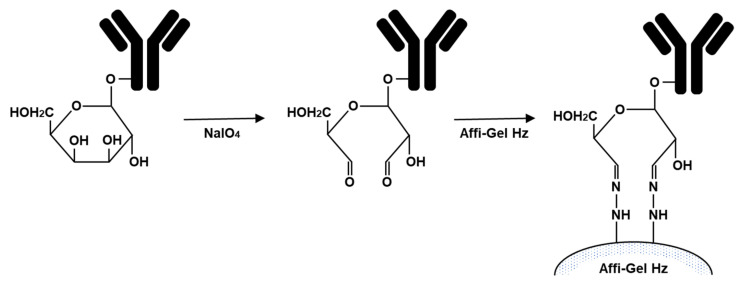
Scheme of the preparation of an immunoaffinity column with MAbs against natural compounds.

IgG contains approximately 3% carbohydrate in the Fc region (heavy chain) of the antibody. Periodate oxidation of vicinal hydroxyl groups of these carbohydrates forms aldehyde group, which can be coupled to Affi-Gel Hz hydrazide gel (Bio-Rad) to form stable hydrazones. The purified MAb was treated with NaIO_4_, leading to the formation of a dialdehyde group on the sugar moiety, and the oxidized MAb was coupled with the gel to form a hydrozone-type immunoaffinity gel (Figure 1) [15,26,47]. In the following sections, we introduce immunoaffinity columns with MAbs against ginsenoside Rb1 and glycyrrhizin, and their clinical application.

Ginseng, the root of *Panax ginseng*, has been used for thousands of years in China, Korea, and Japan as an important crude drug in traditional Chinese medicine and Japanese Kampo medicine. Ginsenosides are the main pharmacologically active compounds in ginseng. Basic and clinical studies have demonstrated that ginsenosides exert various pharmacological activities, such as antioxidative and anticancer effects, which improves immunity, energy and sexuality, cardiovascular diseases, diabetes, and neurological diseases [64]. Ginsenosides are a special group of triterpenoid saponins that can be classified into two groups: protopanaxadiol (PPD) and protopanaxatriol (PPT), which possess a dammarane skeleton in their molecules. To date, more than 150 ginsenosides have been isolated from the roots, leaves, stems, fruits, and flower heads of ginseng [64,65]. Ginseng contains many ginsenosides with similar structures; thus, the separation of specific ginsenosides from ginseng crude extract is difficult. Preparative HPLC can separate and purify individual ginsenosides from crude extracts. However, the disadvantages of preparative HPLC are the cost of the stationary columns, volume of solvents, and run times. In addition, it is difficult to separate specific compounds from the crude extract and finally prepare the specific compound-eliminated crude extract.

**Figure 2:**
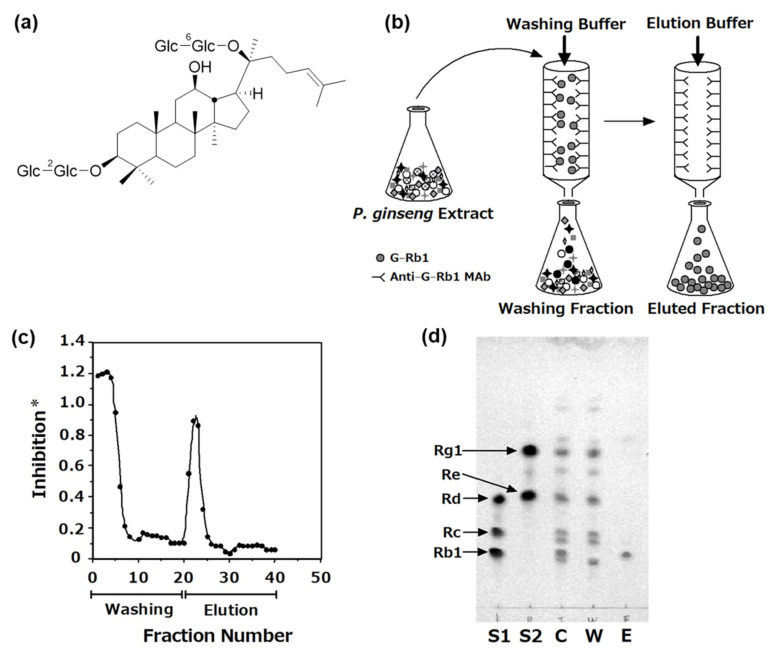
(**a**) Chemical structure of G-Rb1. (**b**) Scheme showing the fractionation using the anti-G-Rb1 MAb-coupled immunoaffinity column. (**c**) Elution profile of the *P. ginseng* crude extract separated using the immunoaffinity column. ELISA using anti-G-Rb1 MAb determined the concentrations of G-Rb1 in each fraction (1–40). * Inhibition = (A0 − A)/A0, where A0 is the absorbance in the absence of the test compounds and A is the absorbance in the presence of the test compounds. (**d**) TLC profile of the fractions obtained from the immunoaffinity column. S1: G-Rd, G-Rc, and G-Rb1, S2: G-Rg1 and G-Re, C: *P. ginseng* crude extract, W: washing fraction, E: eluted fraction.

Ginsenoside Rb1 (G-Rb1, Figure 2a) is a PPD-type ginsenoside, and we established anti-G-Rb1 MAb and performed ELISA [19,20]. The cross-reactivities of MAb with other PPD-type ginsenosides Rc and Rd were 0.024% and 0.020%, respectively, compared to that with G-Rb1 [19,20]. Thus, MAb may predominantly react with G-Rb1 but not with its structurally related ginsenosides. We prepared an immunoaffinity column by coupling MAb with an Affi-Gel Hz gel [26]. The purified 10 mg of IgG diluted in Affi-Gel Hz coupling buffer (Bio-Rad, commercially available) was dialyzed against the coupling buffer. One microliter of the IgG solution was reacted with 100 mL of NaIO_4_ solution (25 mg/1.2 mL H_2_O), and stirred at room temperature for 1 h in a container covered with foil. To inactivate NaIO_4_, glycerol was immediately added to the mixture at a final concentration of 20 mM and stirred for 10 min. The mixture containing the oxidized MAb was dialyzed against the coupling buffer again. After the Affi-Gel Hz hydrazide gel was washed with the coupling buffer, the oxidized and desalted anti-G-Rb1-MAb was then coupled with the gel for 24 h with gentle stirring at room temperature for 24 h. After completing the coupling reaction, the gel was slurry-poured into a mini-column. The column was washed with 20 mM of phosphate buffer (PB) containing 0.5 M NaCl (pH 7.0). The eluates were collected and used for the determination of coupling efficiency by direct ELISA [19]. Finally, the column was washed with phosphate buffer saline (PBS) until the ELISA value was equal to the background, then the column in PBS containing 0.02% sodium azide was stored at 4 °C until ready for use.

The crude extract of *P. ginseng* (3.8 mg) was dissolved in loading buffer (PBS), applied to the immunoaffinity column, and completely washed with the washing buffer (PBS), and then eluted with 100 mM HOAc buffer containing 0.5 M KSCN and 20% MeOH (pH 4.0) (Figure 2b). Figure 2c shows the elution profile of the anti-G-Rb1 immunoaffinity column with anti-G-Rb1 MAb using ELISA. The concentrations of G-Rb1 in each fraction could be easily monitored by this system, so we can know when the column was completely washed. The overcharged G-Rb1 was detected in fractions 1–8. G-Rc, Rd, Re, and Rg1 were also present in these fractions. After washing the column, G-Rb1 was eluted with the elution buffer and detected around fractions 20–24. However, few malonyl G-Rb1 moieties were contaminated in fractions 20–24 because anti-G-Rb1 MAb also reacted with malonyl G-Rb1. Thus, the eluted fraction was treated with a mild alkaline solution at room temperature for 1 h to obtain pure G-Rb1. The washing fractions containing overcharged G-Rb1 were repeatedly applied to the same columns, and G-Rb1 was finally separated from the crude extract. These data demonstrated that G-Rb1, as target compounds, are stable during the separation process. This anti-G-Rb1 immunoaffinity column had the capacity for approximately 20 μg of G-Rb1/mL of gel [26].

After collection, the washing and eluted fractions were deionized and lyophilized. Figure 2d shows the TLC profiles of each fraction. A standard of ginsenosides was spotted on S1 (G-Rd, G-Rc, and G-Rb1) and S2 (G-Rg1 and G-Re). All spots of ginsenosides were detected in the crude extract (C). By contrast, the washing fraction (W) contained all ginsenosides except G-Rb1, suggesting that the column selectively eliminated G-Rb1 from the crude extract. Furthermore, G-Rb1 was detected in the eluted fractions (E). Due to the washing fraction only containing G-Rb1 from the crude extract, this fraction was referred to as the G-Rb1-knockout (KO) ginseng extract [26]. These data indicate the specificity and high efficiency of the immunoaffinity column for eliminating the target compound from the crude extract.

Licorice, the root of *Glycyrrhiza* sp., has long been used worldwide as an herbal medicine. Licorice is registered in the United States Pharmacopoeia 43rd Edition [66], and it is registered as Glycyrrhiza in the Japanese Pharmacopeia 17th Edition (JP XVII) [67]. Licorice is used in more than 70% of Japanese Kampo medicines for the treatment of sore throats. Licorice is also used as a natural sweetener for food and confectionery. The accumulated data on biological activity suggest that licorice exerts antibacterial, antiviral, anti-inflammatory, anticancer, antioxidant, liver protection, neuroprotection, skin whitening, hypoglycemic, memory-enhancing, and other biological activities [68,69,70,71].

**Figure 3:**
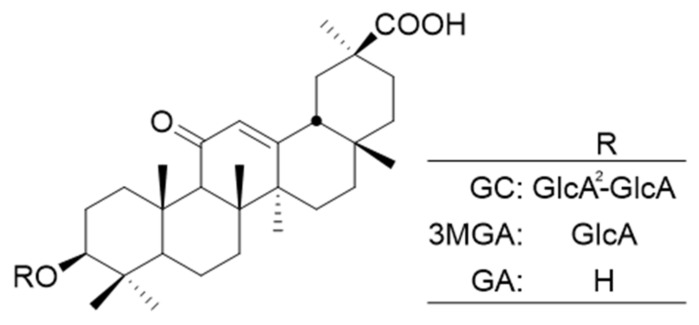
Chemical structures of GC, 3MGA, and GA.

Numerous phytochemical studies have shown that licorice contains numerous bioactive components, including more than 20 triterpenoids and nearly 300 flavonoids [72]. GC (Figure 3), also known as glycyrrhizic acid, is a major bioactive component of licorice and belongs to the triterpene saponin. GC possesses many pharmacological properties, such as antibacterial, antitumor, antiviral, anti-inflammatory, and immunostimulatory activities [73]. In licorice crude extract, GC is present in the highest amount (>2%), and its content in wild, high-quality licorice can be as high as 7% [74]. We previously established anti-GC MAb and reported its application using ELISA and Eastern blotting [13,14]. The cross-reactivity of anti-GC MAb with 3-monoglucuronyl-glycyrrhetinic acid (3MGA) and glycyrrhetinic acid (GA) was 0.585% and 1.865%, respectively. The cross-reactivity of anti-GC MAb with other related compounds including deoxycholic acid, ursolic acid, and oleanolic acid was <0.005%. Thus, ELISA and Eastern blotting, which we established, specifically reacted with GC [13]. To identify high-GC-producing plants, we screened 1025 samples of *G. uralensis* root using a combination of ELISA and Eastern blot and found that the highest concentration of GC was 5.36 dw% among the screened samples [16].

Similar to anti-G-Rb1 MAb, anti-GC MAb was coupled with an Affi-Gel Hz gel to prepare an immunoaffinity column, which can extract GC from the licorice crude extract [15]. The purified 50 mg anti-GC MAb was coupled with 25 mL of an Affi-Gel Hz gel. In the same way as the G-Rb1, anti-GC MAb oxidized with NaIO_4_ by mixing gently in the dark for 1 h, then glycerol was added for the inactivation of NaIO_4_. After dialysis, the oxidized anti-GC-MAb was coupled to the Affi-Gel Hz hydrazide gel for 24 h with gentle stirring at ambient temperature. The gel was poured into a plastic mini-column and the column was washed with 20 mM PB containing 0.5 M NaCl (pH 7.0). The eluates were collected and saved for coupling efficiency determination. The coupling efficiency of the anti-GC-MAb to Affi-Gel Hz was determined to be 95.21% using a sandwich ELISA to measure uncoupled MAb.

**Figure 4:**
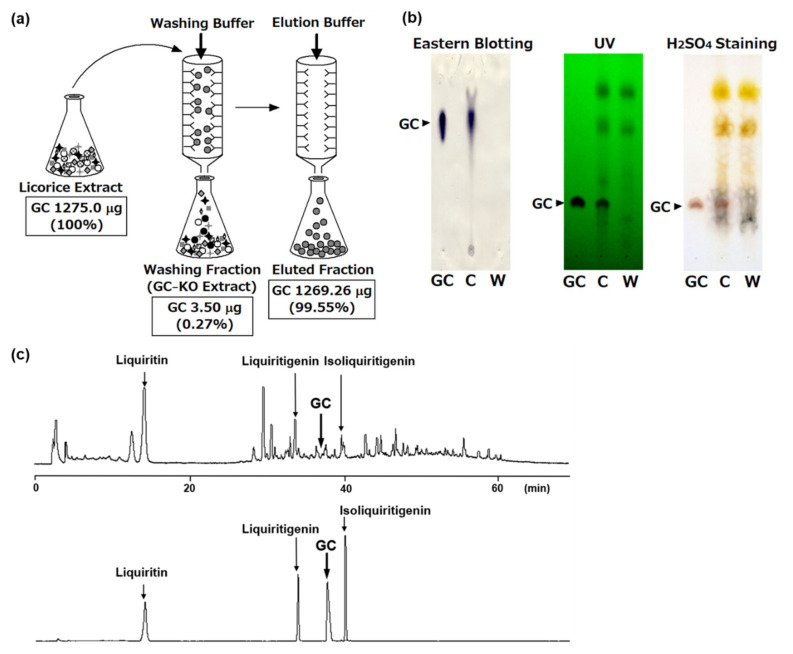
(**a**) Scheme of the preparation of GC-KO extract using the anti-GC MAb-coupled immunoaffinity column and the GC concentration in each fraction. (**b**) TLC profiles of the fractions obtained from the immunoaffinity column. C: licorice crude extract; W: washing fraction. (**c**) HPLC profile of the washing fraction (upper panel), GC, and three licorice flavonoids (lower).

This affinity column specifically and completely eliminated GC from the crude licorice extract. Twelve milligrams of crude extract containing 1275.0 μg of GC was dissolved in loading buffer (5 mM PB–5% MeOH–50 mM NaCl, pH 7.0) and applied to the immunoaffinity column. The loading buffer was mechanically circulated through the column to enhance the binding efficiency of GC. After overnight circulation, the unbound fraction was collected. The column was then washed with washing buffer (20 mL of 5 mM PB–50 mM NaCl, pH 7.0) and eluted with elution buffer (20 mM PB–30% MeOH–500 mM NaCl, pH 7.0). After deionization and lyophilization of the solvent in each fraction, the GC concentration was determined using ELISA. The GC concentrations in the unbound and bound fractions were 3.50 μg (0.27% of the applied GC) and 1269.26 μg (99.55% of the applied GC), respectively (Figure 4a). In other words, the anti-GC immunoaffinity column eliminated 99.55% of the loaded GC and the capacity of the anti-GC immunoaffinity column was approximately 33.5 μg of GC/mL of gel. The obtained unbound fraction was the licorice crude extract lacking only GC; therefore, this unbound fraction had the GC-KO licorice extract. TLC analysis of the GC-KO licorice extract indicated that GC was specifically removed from the GC-KO licorice extract (Figure 4b). In addition, eastern blotting using anti-GC MAb could not detect GC in the GC-KO licorice extract. HPLC fingerprint analysis demonstrated that although the GC-KO licorice extract contained three licorice flavonoids, namely, liquiritin, liquiritigenin, and isoliquiritigenin, in the same pattern as that in the licorice extract, only GC was undetectable in the GC-KO licorice extract (Figure 4c).

In order to determine the purity of target compounds, we analyzed the eluted fractions by ELISA analysis and Eastern blotting, suggesting that the eluted fractions contained only target compounds bound to MAbs. Although the preparation of the immunoaffinity columns consumes a large amount of MAbs, the column is reusable after cleaning. The column capacity was stable even after more than 10 purification cycles under the same conditions, similar to the one-step separation of forskolin present in the crude extract of *Coleus forskohlii* root [10]. According to the ELISA analysis of eluted fraction from the anti-GC immunoaffinity column, this column could be regenerated in excess of 20 times without an obvious loss of capacity (from about 33 μg/mL gel to about 29 μg/mL gel) [15]. Generally, the purification and separation of natural compounds have been performed by HPLC and chromatographic techniques, but these are tedious and time-consuming procedures. Compared with these conventional methods, the methodology using the immunoaffinity column is simple and specific. In addition, once the column is prepared, it is reusable several times without a loss in activity. Recently, molecular imprinted polymers (MIP) have attracted wide attention and attained significant applications in the identification and separation of various natural compounds [75]. MIP is useful tool to isolate natural compounds, but MIP applications in the analytical determination of natural compounds such as polyphenols metabolites have been limited and few used phenolic acid as a template [76]. In addition, the preparation of KO extracts using MIP has never been reported before. Thus, the immunoaffinity column with MAbs against natural compounds is only tool to prepare KO extracts.

## 3. Cell-Based Studies Using the GC-KO Licorice Extract

The KO extract is a useful tool for evaluating the interaction or synergistic actions between the target compound and other compounds present in the crude extract. In this section, we present two previous reports that have performed cell-based studies using the GC-KO licorice extract.

The first example of a study using the GC-KO licorice extract is to clarify the role of GC in the suppression of nitric oxide (NO) production [77]. The licorice extract suppressed NO production in lipopolysaccharide-treated murine RAW264 macrophages (inhibition ratio (IR) = 57.7%). On the contrary, single treatment of GC could not significantly inhibit NO production. Therefore, it seems that the GC-KO licorice extract can inhibit NO production to a level similar to that of the licorice extract because single treatment of GC did not inhibit NO production. Interestingly, although GC alone did not block NO production, the inhibitory effect of the GC-KO extract (IR = 17.8%) was weaker than that of the licorice extract (IR = 57.7%). Moreover, co-treatment with the GC-KO extract and GC strikingly improved the inhibitory potency (IR = 33.5%). A similar pattern was found for the protein expression levels of inducible NO synthase, which produces NO from L-arginine. These data indicate that GC alone cannot attenuate NO production, but GC suppresses NO production with the other constituents of licorice. Moreover, a similar phenomenon was observed when we compared the antiproliferative activity and apoptosis induction of licorice extract and GC-KO extract on antiproliferative activity and apoptosis induction in human leukemia HL-60 cells [78]. These data imply the synergistic interactions of GC and other constituents in the licorice extract.

In the second study, GC-KO licorice extract was used to explore the effect of licorice extract on high glucose-induced tubular epithelial-to-mesenchymal transition (EMT) [79]. In renal tubular NRK-52E cells exposed to high-glucose, GC-KO extract provided equivalent efficacy compared with that of the licorice extract in suppressing EMT via Notch2 signaling pathway. By contrast, GC had little influence on EMT. Although GC is a major bioactive component, licorice extract contains various other bioactive components [69,72]. Thus, these data indicated that GC was not directly involved in the suppression of EMT, and both licorice and GC-KO extracts could prevent renal tubular EMT and fibrosis in diabetic nephropathy. Future studies are needed to elucidate the role of GC on EMT.

The two previous studies demonstrated that the KO extract is helpful for identifying the potential role of target compounds in crude extract in cell-based studies. Similar to the G-Rb1-KO extract and the GC-KO extract, several immunoaffinity columns and KO extracts were reported by our group and other groups (Table 1) [60,61,62]. The selectivity of the column is dependent on the MAbs conjugated with Affi-Gel Hz gel. The MAb against solasodine has wide cross-reactivity. The immunoaffinity column coupled with anti-solamargine MAb could separate total solasodine glycosides from the crude extract of *Solanum khasianum* seeds [46]. In other words, this column can concentrate the total solasodine glycosides from plant materials. Therefore, this methodology can be available for the detection of higher yielding solasodine glycosides plantlets in vitro of *S. khasianum* by the combination with ELISA because the regenerated plantlets contain a small amount of solasodine glycosides. Qu et al. prepared the immunoaffinity column by coupling the MAbs against daizin and naringin to CNBr-activated Sepharose 4B. The immunoaffinity column coupled with anti-daizin MAb can efficiently and specifically extract daidzin, glycitein, and genistin from numerous structurally similar soy isoflavones in leguminous plants [61]. On the other hand, the column coupled with anti-naringin MAb can capture approximately 250 μg of naringin without cross reacting with its structurally similar compounds [62]. Taken together, these studies imply that various immunoaffinity column having different cross-reactivity against natural compounds can be established and applied for the preparation of KO extracts.

## 4. Conclusions

This review discusses the one-step purification of bioactive natural compounds from crude extracts using an immunoaffinity column coupled with MAbs against natural compounds. G-Rb1 and GC were selectively separated from *P. ginseng* and licorice crude extract by the column using MAbs against G-Rb1 and GC, respectively. The obtained washing fractions were eliminated only target compound from the crude extract; therefore, this fraction is referred to as KO extract. This immunoaffinity column selectively separated target compounds from the original extract without time-consuming and complicated procedures. Once the column is prepared, the column is stable and reusable for more than 10 cycles under the same conditions. This methodology may be applied to other kinds of natural compounds not yet been developed. The selectivity of the column is dependent on the MAbs, thus when the column is coupled with a broad cross-reactive MAb, it is possible to separate a group of target compounds, not only one kind of compound, from the extract. Indeed, we demonstrated that total ginseng saponins were separated by the column using anti-G-Re MAb, which has a broad cross-reactivity with ginsenosides [29]. The immunoaffinity columns can be applicable for a high-sensitivity detection of the target compounds by combination with ELISA. In our previous study, the combination of column and ELISA using anti-G-Rb1 detected G-Rb1 in *Kalopanax pictus* Nakai, which was not previously reported to contain ginsenosides [23]. We also applied KO extract for investigating the functions of natural compounds in cell-based studies. The KO extracts may be useful tool to clarify the actual effects of the bioactive compound and elucidate interactions between the target compound and the other compounds in the crude extracts, including Japanese Kampo medicines and traditional Chinese medicines.

## Figures and Tables

**Table 1:** Example of applications using MAbs against natural compounds.

Target Compound	Plant Resource	Applications	References
**Monoterpene**			
Paeoniflorin, Albiflorin	*Paeonia lactiflora*	ELISA	[3]
		Immunostaining of plant section	[4]
**Sesquiterpenes**			
Artemisinin, Artesunate	*Artemisia annua*	ELISA	[5]
**Diterpene**			
Paclitaxel	*Taxus* sp.	ELISA	[6,7]
		Time-resolved fluoroimmunoassay	[8]
Forskolin	*Coleus forskohlii*	ELISA	[9]
		Immunoaffinity column	[10]
**Triterpene**			
Glycyrrhizin	*Glycyrrhiza* sp.	ELISA	[11,12]
		Eastern blot	[13]
		Double eastern blot	[14]
		Immunoaffinity column	[15]
		Selective breeding	[16]
3-Monoglucuronyl-glycyrrhetinic acid	*Glycyrrhiza* sp.	ELISA	[17]
		Immunodetection in plasma and urine of patients	[17]
		Eastern blot	[18]
Ginsenoside Rb1	*Panax* sp.	ELISA	[19,20]
		Immunodetection in rat serum	[21,22]
		Eastern blot	[23]
		Double eastern blot	[24,25]
		Immunoaffinity column	[26]
		Cellular localization	[27]
Ginsenoside Rg1	*Panax* sp.	ELISA	[20,28]
		Immunodetection in rat serum	[21]
		Double eastern blot	[24,25]
Ginsenoside Re	*Panax* sp.	ELISA	[20,29]
		Eastern blotting	[30]
		KO extract	[31]
Notoginsenoside R1	*Panax notoginseng*	ELISA	[32]
Saikosaponin a	*Bupleurum falcatum*	ELISA	[33,34]
		Time-resolved fluoroimmunoassay	[35]
**Tetraterpene**			
Crocin	*Crocus sativus*	ELISA	[36]
**Chroman**			
Tetrahydrocannabinolic acid	*Cannavis sativa*	ELISA	[37]
**Quinone**			
Sennoside A,	*Rheum sp.,*	ELISA	[38]
Sennoside B	*Senna sp.*	Eastern blotting	[39]
Plumbagin	*Plumbago zeylanica*	ELISA	[40,41]
		Molecular breeding	[42]
**Alkaloid**			
Berberine	*Phellodendron amurense* *Coptis japonica*	ELISA	[43,44]
Solamargine	*Solanum khasianum*	ELISA	[45]
		Eastern blotting	[46]
		Immunoaffinity column	[47]
		Molecular breeding	[48]
Aristolochic acid-I, -II	*Aristolochia* species	ELISA	[49]
		Eastern blot	[50]
		Cellular localization	[51]
		Determination of target molecular	[52]
Harringtonine	genus *Cephalotaxus*	ELISA	[53]
		Immunochromatographic strip assay	[54]
		Cellular uptake	[55]
**Flavonoid**			
Liquiritin	*Glycyrrhiza* sp.	ELISA	[12,56]
		Double eastern blot	[14]
		Quality control	[56]
Baicalin, Baicalein	*Scutellaria baicalensis*	ELISA	[57]
Kwakhurin	*Pueraria candollei* var. *mirifica*	ELISA	[58,59]
Puerarin	*Pueraria lobata*	ELISA	[60]
		Immunoaffinity column	[60]
Daidzin	*Glycine max*	ELISA	[61]
		Immunoaffinity column	[61]
Naringin	*Citrus* sp.	ELISA	[62]
		Immunoaffinity column	[62]

## Data Availability

Data in this study will be provided upon reasonable request to the corresponding author.
